# Probucol promotes osteoblasts differentiation and prevents osteoporosis development through reducing oxidative stress

**DOI:** 10.1186/s10020-022-00503-7

**Published:** 2022-06-28

**Authors:** Zhou-Shan Tao, Tian-Lin Li, Shan Wei

**Affiliations:** 1 Department of Orthopedics, The First Affiliated Hospital of Wannan Medical College, Yijishan Hospital, No. 2, Zhe Shan Xi Road, Wuhu, 241001 Anhui People’s Republic of China; 2 School of Mechanical Engineering, Anhui Polytechnic University, Wuhu, 241000 People’s Republic of China; 3 Additive Manufacturing Institute of Anhui Polytechnic University, Anhui Polytechnic University, Wuhu, 241000 People’s Republic of China

**Keywords:** Osteoporosis, Osteogenesis, Oxidative stress, Bone metabolism, Probucol

## Abstract

Probucol (PBC) is a potent cholesterol-lowering drug and has been studied extensively for its powerful antioxidative stress. The purpose of this study is to investigate the role of PBC in ovariectomized rat model and to explore the mechanism of osteogenic differentiation of MC3TE-E1 Cells. RT-qPCR and Immunofluorescence were used to measure the expression level of SOD2, SIRT1, intracellular oxidative stress levels and osteogenic markers proteins. Moreover, CCK-8 assay was conducted to detect cell viability. Alizarin red staining and alkaline phosphatase staining were applied to examine osteogenic function and calcium deposits. The ovariectomized rat model was set up successfully and HE staining were employed to examine femoral trabeculae tissue. Our results showed that PBC suppressed MC3TE-E1 resist oxidative stress to promote osteogenic differentiation. Additionally, it was confirmed that PBC promoted osteogenic differentiation of MC3TE-E1 through inhibiting oxidative stress. Further study indicated that PBC exerted its beneficial function by suppressing oxidative stress-mediated alter bone metabolism to alleviate osteoporosis in vivo. Our research suggested that the PBC-modulated oxidative stress inhibition is responsible for activation of the process of osteogenic differentiation, providing a novel insight into the treatment of osteoporosis.

**Graphical abstract:**

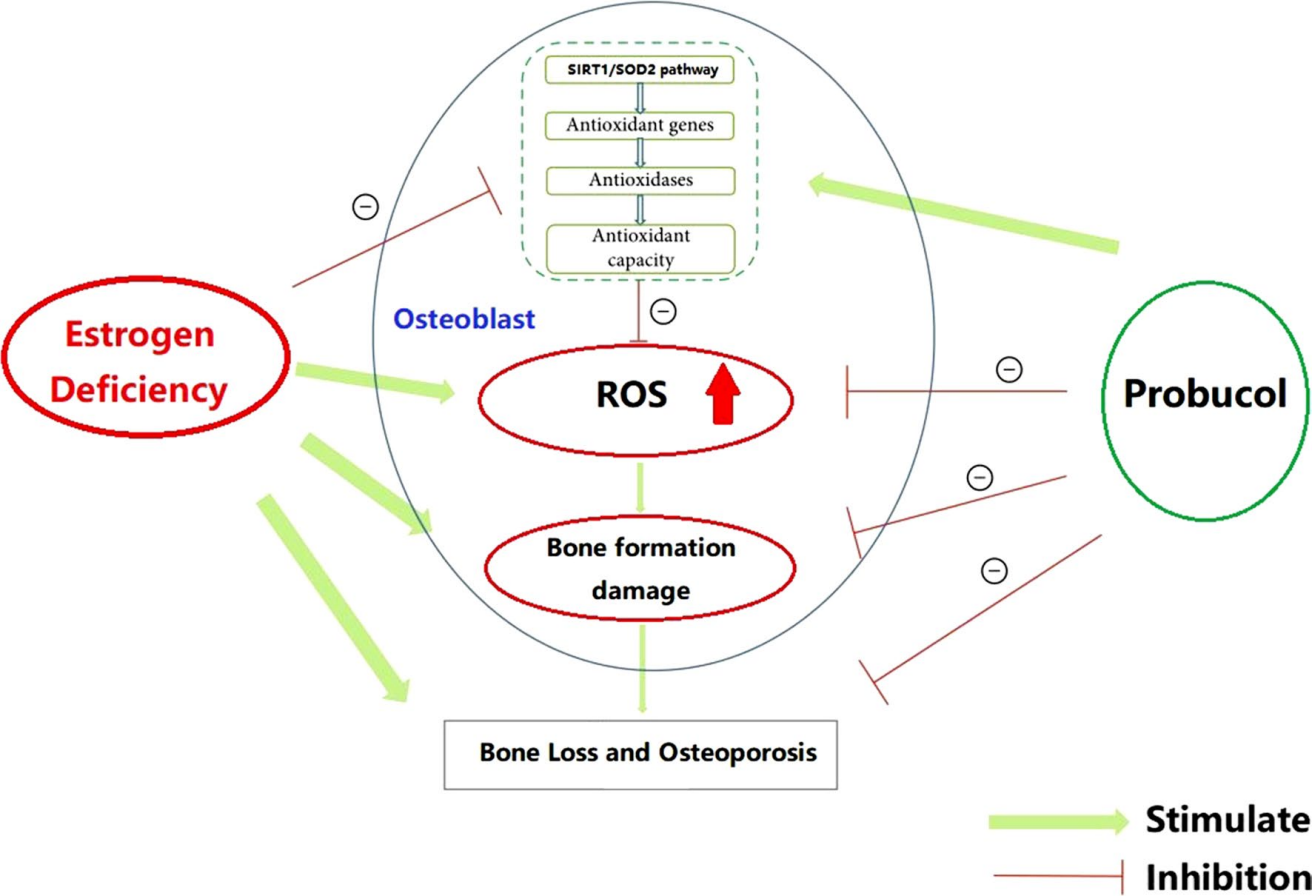

## Introduction

Osteoporosis is a systemic bone illness characterized by declined bone strength, bone mass, and elevation of deterioration of microstructure and brittleness, making patients to be more prone to fracture. Estrogen absence results in postmenopausal osteoporosis, inducing elevated bone metabolism and ultimately forfeiting bone trabecular connectivity and cortical osteoporosis (Ma et al. 2019). It is estimated that the number of women over the age of 50 with osteoporosis is about twice that of men (Kim et al. 2019). Its pathogenesis involves dysregulated interactions between osteoblasts and osteoclasts, leading to bone formation and resorption imbalance (Cheng et al. 2019). Many works suggest that oxidative stress plays a pivotal role in the development and progression of various types of osteoporosis (Munmun and Witt-Enderby 2021; Tao et al. 2022). The level of oxidative stress will increase with osteoporosis deterioration, which indicates that oxidative stress plays a critical role in the progression of bone loss (Zhou et al. 2019). Moreover, higher oxidative stress not only induces dramatic apoptosis in primary osteoblasts, but more importantly inhibits osteogenic differentiation (Baek et al. 2010). In addition, antioxidant drugs such as melatonin, alpha lipoic acid and resveratrol have beneficial effects on the differentiation and mineralization functions in osteoblast, as well as treatment of osteoporosis (Lu et al. 2017; Chen et al. 2022; Liu et al. 2021).

Probucol (PBC) is a cholesterol-lowering agent, but it has been receiving increasing attention for multiple reasons, including immunomodulation, anti-oxidation and neuroprotection (Iqbal et al. 2004). Studies have reported that PBC could have a crucial role in preventing ethanol-induced liver fibrosis, treatment of erectile dysfunction and renal disease through inhibiting lipid peroxidation and oxidatively modifying low-density lipoprotein particles in vitro, circumnavigates endothelial impairment (Su et al. 2014; Jiang et al. 2002; Wang et al. 2020). Interestingly, some studies demonstrated that PBC is also protective in experimental models of neurotoxicity/neuropathology (Colle et al. 2013). Owing to reactive oxygen species (ROS) playing a pivotal role in the occurrence of different types of osteoporosis, PBC may prevent bone loss in OVX rats by reversing oxidative stress-induced damage to bone formation for strong anti-oxidative stress ability. However, to our knowledge, the role of PBC in ovariectomy (OVX)-induced osteoporosis and osteoblast-mediated bone formation remains unclear.

Therefore, it’s worth further defining the effects of PBC on osteoporosis. In our study, we tested the hypothesis that PBC prevents estrogen deficiency-induced bone loss and protects osteoblasts from oxidative stress, and tried to provide new evidence for using PBC on the prevention and treatment of osteoporosis.

## Materials and methods

### Culture of osteoblasts

MC3T3-E1 cells were obtained from Qingqi (Shanghai) Biotechnology Development Co., Ltd.) in China and cultured in the minimum necessary medium α (Gibco) supplemented with 10% fetal bovine serum, 100 U/mL penicillin and 100 μg/mL streptomycin under 37 ℃, 5% CO_2_ circulation cell culture incubator. In addition to cell cultures, appropriate amount of PBC (Sigma-Aldrich, St. Louis, Mo, USA) powder was dissolved in PBS, and the concentration of the drug was mixed into a 10^–4^ mol/L storage solution, which was filtered for sterilization and stored for later use. When we used each, it was diluted to 0.1 µmol/L by mixing 10% fetal bovine serum (Bovogen, New Zealand, Australia) and DMEM medium (Gibco, New York, USA).

### Osteoblast viability measurement

Detection of cell viability was via the cell counting kit (CCK)-8 method. Seeding of MC3T3-E1 cells was into 96-well plates at a density of 5 × 10^3^ cells/well, and incubation for 24 h, 48 h, and 72 h under the following three conditions: DMEM (Control), DMEM + 30 μM H_2_O_2_ (H_2_O_2_), DMEM + 1 µM PBC + 30 μM H_2_O_2_ (1 µM PBC + H_2_O_2_) and DMEM + 10 µM PBC + 30 μM H_2_O_2_ (10 µM PBC + H_2_O_2_). The concentration of probucol (Sigma, St. Louis, MO, USA) was determined based on the published in vitro studies on probucol (Li et al. 2021a). Then, 10 mL CCK-8 reagent was in each well. Measurement of the absorbance was at 450 nm with a microplate reader. Performance of cytotoxicity tests was evaluated with lactate dehydrogenase (LDH) detection kits (Nanjing Jiancheng, Nanjing, China) in line with the manufacturer’s agreement.

### Intracellular oxidative stress measurement and immunofluorescence

To measure the levels of intracellular ROS, MC3TE-E1 was incubated with 10 μM of 2,7-dichlorofluorescein diacetate (DCFH-DA, Beyotime) for 30 min at 37 °C. SIRT1 and SOD2 expression in the cells was evaluated by immunofluorescence staining. After the cells were cultured for 24 h, the cells were fixed in 4% paraformaldehyde solution at room temperature for 15 min. and washed three times with PBS. Then SIRT1 and SOD2 antibodies were added for incubation overnight at 4 °C. After rinsing with PBS, the cells were incubated with fluorescent secondary antibody at room temperature for 2 h. After rinsing with PBS for three times, nuclei were counterstained by addition of 5 µg/mL DAPI staining solution for 5 min. Finally, the expression of SIRT1 and SOD2 was observed in each group of cells.

### Alkaline phosphatase (ALP) staining and alizarin red-s (ARS) staining

For assessment of osteogenic differentiation based on alkaline phosphatase (ALP) production, cells of the five groups were cultured in 24-well plates for 14 days. For ALP measurement, the cells were first fixed in 4% paraformaldehyde solution at room temperature for 15 min. Then NBT/BCIP ALP reagent (Beyotime, Shanghai, China) was applied for 30 min for ALP staining. The cells were then rinsed with phosphate-buffered saline (PBS) three times before observation of ALP staining. For normalization, an alkaline protease (AKP) kit (Beyotime, Shanghai, China) was used to assess protein activity, and the data for each group were obtained by a microplate reader.

For assessment of osteogenic differentiation based on the formation of calcium deposits, cells of the five groups were cultured in 24-well plates for 21 days. Cells in each well were first fixed at room temperature in 4% paraformaldehyde solution for 15 min. The cells were then stained with 1% Alizarin Red S (Beyotime, Shanghai, China) for 20 min and washed three times with PBS for observation of calcium deposition. The percentage area in each well that was Alizarin Red S positive was analyzed using Image-J software (NIH).

### RNA extraction and real-time PCR

After the cells were cultured for 2 h, Gene expression levels in cells were assessed by real-time quantitative polymerase chain reaction (PCR) analysis. First, total RNA was extracted from each group of cells using the Trizol reagent, and cDNA was reverse transcribed into cDNA by applying a cDNA reverse transcription reagent. Real-time PCR was performed using SYBR^®^ Green Realtime PCR Master Mix, and PCR products were detected using the StepOnePlus Realtime PCR System. GAPDH was selected as the internal reference gene. A total of 40 cycles were performed according to the reaction procedure of 95 °C, 1 min; 95 °C, 15 s; 60 °C, 15 s; and 72 °C, 45 s. Melting curve analysis was performed after the reaction was completed. The primers used in this study were listed in Table 1.

**Table 1:** Primer sequences for the real-time polymerase chain reaction of the genes associated osteoblast differentiation

Target gene	Forward primer (5ʹ-3ʹ)	Reverse primer (5ʹ-3ʹ)
Runx2	GCCGTAGAGAGCAGGGAAGAC	CTGGCTTGGATTAGGGAGTCAC
OC	CGGCAAGGCTTCGCATCTG	GGAGCAGAGCAGACAGGTGAACT
ALP	AGCGACACGGACAAGAAGC	GGCAAAGACCGCCACATC
SOD1	GGTGGGCCAAAGGATGAAGAG	CCACAAGCCAAACGACTTCC
SOD2	GGGGATTGATGTGTGGGAGCACG	AGACAGGACGTTATCTTGCTGGGA
CAT	TGG GAT CTC GTT GGA AAT AACAC	TCA GGA CGT AGG CTC CAG AAG
GAPDH	AGAAAAACCTGCCAAATATGATGAC	TGGGTGTCGCTGTTGAAGTC

Superoxide dismutase 1 and 2 (SOD1, 2); Glyceraldehyde 3-phosphate dehydrogenase (GAPDH); Osteocalcin (OC); RUNX2 (core binding factor alpha 1, core binding factor a1); Catalase (CAT)

### Experimental animals

Three-month-old female SD rats (n = 50) were obtained from Zhejiang Provincial Laboratory Animal Center (Hangzhou, China). After 1 week of stabilization, rats were randomly divided into four groups; 35 rats underwent bilateral ovariectomy (OVX) surgery and 15 rats were subjected to a Sham operation as previously described (Tao et al. 2016, 2019). Anesthesia of the rats was with pentobarbital sodium (100 mg/kg i.p.) and excision of their ovaries was conducted for construction of the classical postmenopausal osteoporosis rat model. 12 weeks after ovariectomy, rats were sacrificed and femurs were evaluated with imaging and histology to determine the establishment of a hormone-deficiency-induced postmenopausal osteoporosis model. The rats were casually assigned into the Sham group, OVX groups, OVX + LPBC and OVX + HPBC group, with 10 rats each. Then, the Sham group and OVX groups were treated with vehicle reagents, and the OVX + LPBC and OVX + HPBC groups were treated with lower probucol (1 mg/kg body weight per day) and higher probucol (3.5 mg/kg body weight per day) oral gavage. The dosage and mode of probucol used in the experiment for its effective anti-oxidative stress as a previously study reported (Colle et al. 2013). Then all venous blood was taken from the abdominal aorta under anesthesia, and euthanasia was via dislocation of the neck. Right femurs were frozen to measure trabecular parameters, while left ones were preserved in formaldehyde for histological study. All experiments were approved and carried out in accordance with international standards on animal welfare and the Institutional Animal Care Committee of Wannan Medical College (Approval No. LLSC-2020-082).

### Micro-computerized tomography (Micro-CT)

The changes of bone mass were evaluated by micro-CT (micro-CT 100, Scanco Medical AG, Bassersdorf, Switzerland) as described in previous reports (Tao et al. 2021, 2022). Micro-CT scanning was performed at 15 μm resolution, at 70 kV and 200 μA, with an integration time of 300 ms, and remodeled with an isotropic voxel size of 5 mm. 2 mm proximal to femoral epiphysis line were selected as the volume of interest (VOI). The bone morphometric parameters, bone volume per total volume (BV/TV), bone trabecular thickness (Tb.Th), trabecular number (Tb.N), trabecular spacing (Tb.Sp), connective density (Conn.D) and BMD were calculated within the VOI zone.

### Histological analysis and immunohistochemical analysis

Then femurs were placed into a decalcification solution of 10% EDTA and 4% phosphate buffered formalin solution for 28 days at 4 °C. Subsequently, bone tissue was fixed in 10% (v/v) formalin, embedded in paraffin, and sliced into 5 mm-thick sections. HE staining was performed using an HE staining kit (Solarbio, Beijing, China) according to the manufacturer's instructions. After staining the bone tissue, the images were collected with the ECLIPSE Ti-U (Nikon), as previously described (Li et al. 2021b; Tao et al. 2020).

Immunohistochemical analysis was performed using an anti-Osteocalcin (OC) antibody and anti-Tartrate resistant acid phosphatase-5b (TRAP-5b) antibody. Briefly, 3% hydrogen peroxide was used to block endogenous peroxidase, and trypsin was applied to expose the antigen. The sections were irrigated and incubated overnight with the commercially-available specific antibodies OC (1:100, Abcam, Cambridge, UK) and TRACP-5b (1:300, Abcam, Cambridge, UK) at 4 °C. Finally, the slides were incubated with the corresponding goat anti-rabbit secondary antibody for 30 min and counter-stained with diaminobenzidine and hematoxylin. All sections were examined and photographed under a light microscope and analyzed using Image Pro Plus software (Media Cybernetics, Rockville, MD, USA).

### Analysis of bone formation and resorption markers

Serum samples were stored at − 80 °C until final analyses were carried out. Serum PINP (N-terminal propeptide of type I procollagen) levels were measured using a rat competitive enzyme immunoassay (EIA) assay (Immunodiagnostic Systems EURL, Paris, France), according to the manufacturer’s protocol. Serum PINP (N-terminal propeptide of type I procollagen) and type I collagen crosslinked C-telopeptide (CTX-I) levels were measured using a rat competitive enzyme immunoassay (EIA) assay (Beijing Bright Biotechnology Co., Ltd.) and Serum CrossLaps^®^ (CTX-I) ELISA kit (Beijing Bright Biotechnology Co., Ltd.), according to the manufacturer’s protocol.

### Statistical analysis

All data are shown as mean ± standard deviation, analyzed using SPSS ver. 22.0 software (IBM SPSS Statistics for Windows, Armonk, NY, USA). One-way analysis of variance (ANOVA) was used for multiple between-group comparisons followed by Tukey’s post-hoc test. The independent t-test was used for comparisons of normal groups and OVX groups. A value of P ≤ 0.05 was considered to reflect significance.

## Results

### Experimental animal

A total of 3 rats died during or after building a bone defect model, including 1 rat in the OVX group, 1 rat in the OVX + LPBC group died 1 day after surgery and 1 rat in the OVX + HPBC group died 2 days after surgery. The remaining rats went through operative procedures and recovered well, and participated in all subsequent experiments.

### Osteoporosis animal model

**Fig. 1:**
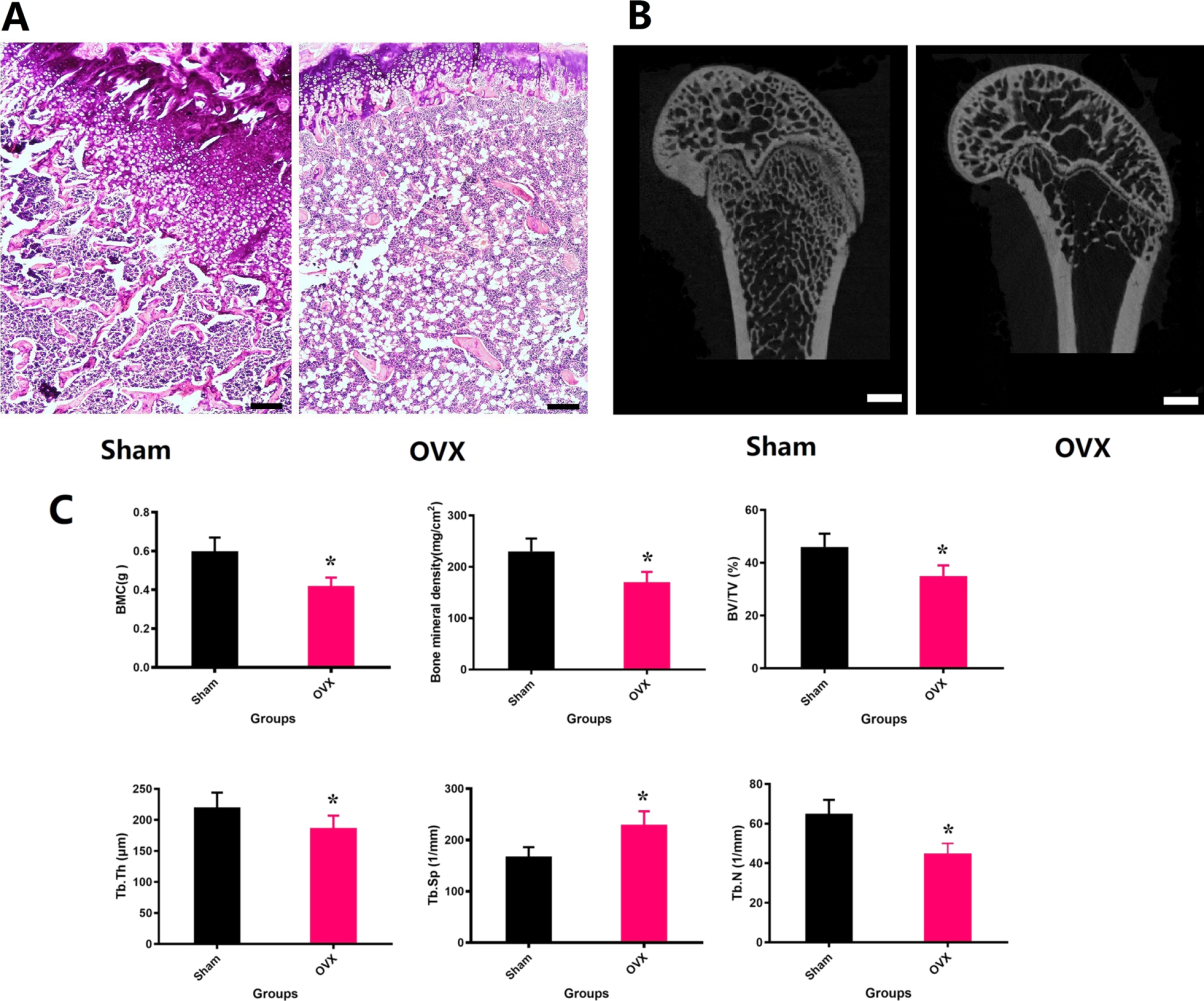
**A** Representative H&E staining for the normal and osteoporotic femur (magnification of 20). **B** 2D micro-CT images of femoral metaphysis in sham and OVX rats, the scale bar represents 1 mm. **C** The BMD, BMC, BV/TV, Tb.N, Tb.Th, and Tb.Sp of trabecular bone of femoral metaphysis in OVX group and sham group. *P < 0.05 versus the Sham group. n = 5 per group

Twelve weeks after the first operation, 5 representative rats in each group were sacrificed and the trabecular architecture of the rat femoral metaphysis was measured by Micro-CT and HE. Figure 1 clearly shows that the trabecular bone in the distal femoral metaphysis significantly decreased in the ovariectomized rats, indicating severe bone loss after surgery. The quantitative results of BMD, BMC, BV/TV, Tb.Th, Tb.N and Tb.Sp are presented in Fig. 1 and shows that there are significant statistical differences in the above-mentioned indexes between the two groups (P < 0.05). When observing HE sections under a microscope, it is easy to find the same results. These results indicate that a bilateral ovariectomy-induced osteoporotic rat model was successfully established.

### Micro-CT evaluation

**Fig. 2:**
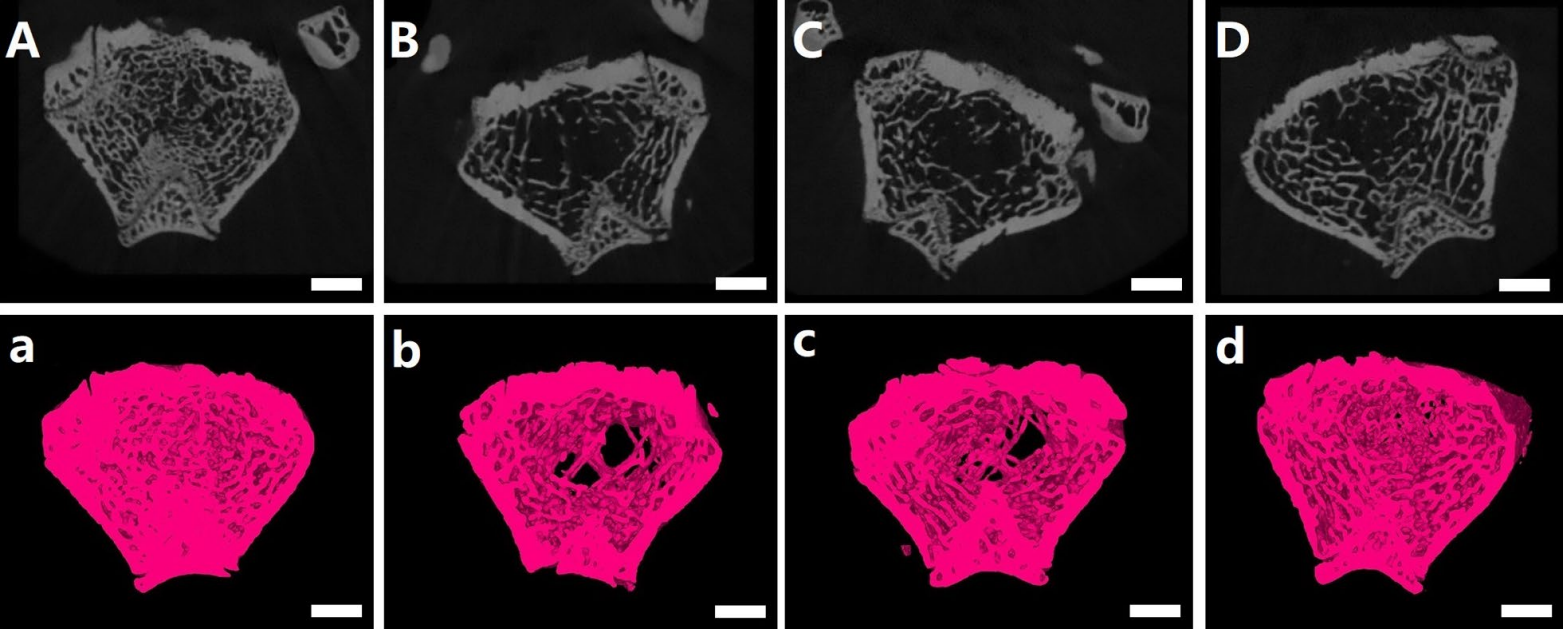
Representative Micro-CT images with 2D and 3D of the changes of femoral metaphysis trabecular bone in the Sham (**A**, **a**), OVX (**B**, **b**), OVX + LPBC (**C**, **c**) and OVX + HPBC (**D**, **d**) groups. The scale bar represents 2 mm

**Fig. 3:**
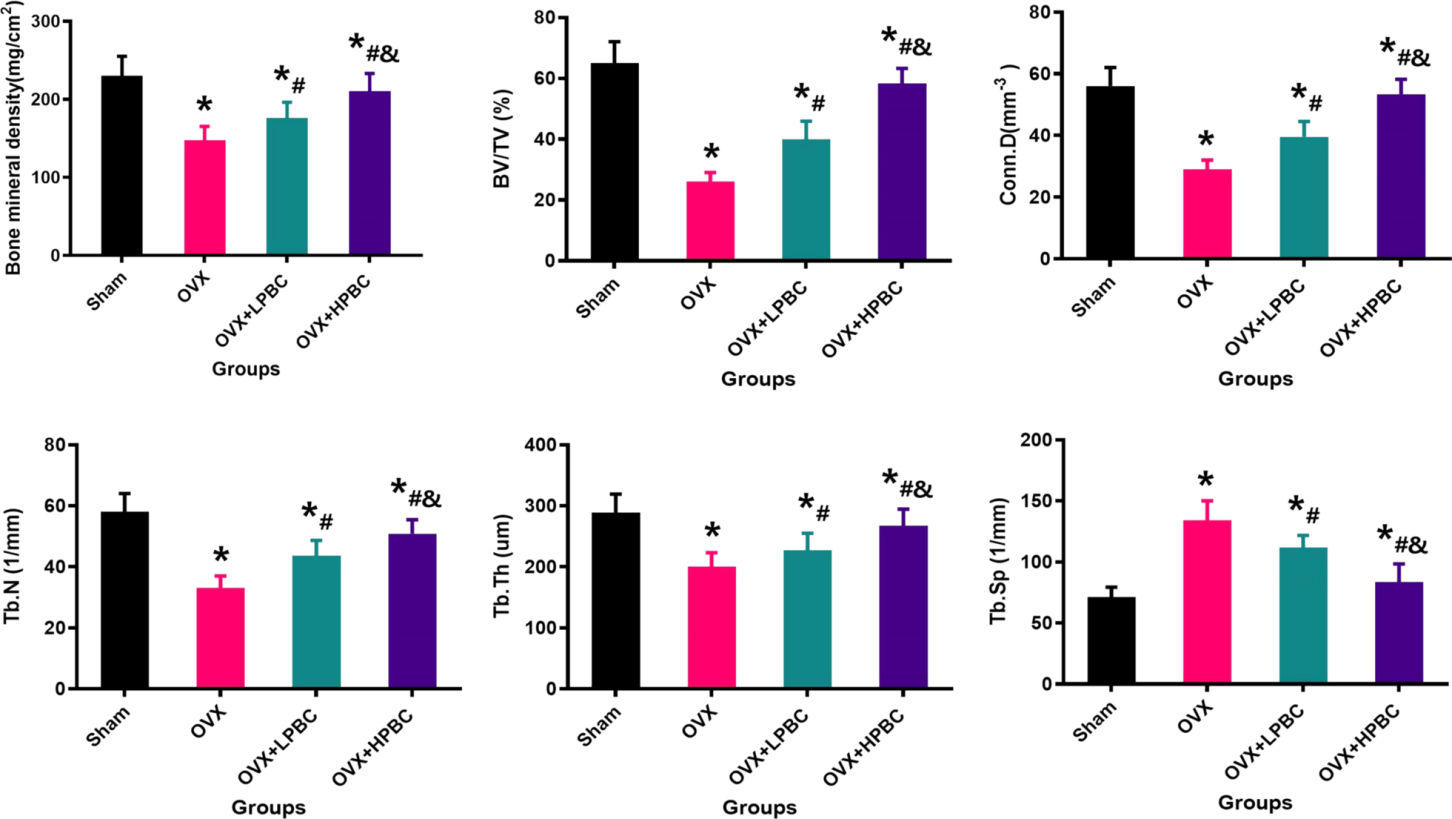
Quantitative results of trabeculae bone in femoral metaphysis including BMD, BV/TV, Tb.N, Conn.D, Tb.Th, and Tb.Sp. n = 5 per group. *Vs. Sham group, P < 0.05, ^#^Vs. OVX, P < 0.05, ^&^Vs. OVX + LPBC group

The 2D scan images (Fig. 2A–D) and 3D reconstruction images (Fig. 2a–d) of Micro-CT clearly shows us the changes of femoral metaphysis trabecular bone after 12 weeks of treatment with different intervention methods. We can easily observe that the trabecular bone of the OVX + HPBC group was almost filled with femoral metaphysis, while large amounts of trabecular bone was found in the OVX + LPBC group, but it was difficult to find the trabecular bone in the OVX group. The quantitative results were expressed as BMD, BV/TV, Tb.Th, Tb.N, Conn.D and Tb.Sp (Fig. 3). Compared to the OVX + LPBC group and the OVX group, higher PBC treatments significantly improved the trabecular bone as measured by these parameters; the increased BMD, BV/TV, Tb.Th, Tb.N, Conn.D and decreased Tb.Sp (P < 0.05).

### Histological analysis

**Fig. 4:**
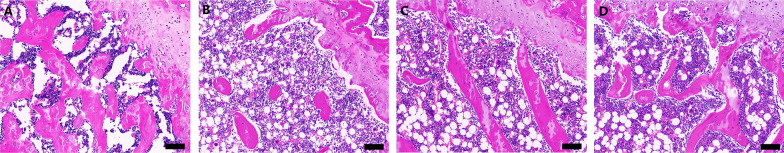
Bone tissue of femoral metaphysis by histological analysis by HE staining in the groups. Representative images of HE staining (**A**–**D)**, magnification (× 20). Sham (**A**), OVX (**B**), OVX + LPBC (**C**) and OVX + HPBC (**D**). n = 5 per group

Histological images with HE staining present, as shown in Fig. 4, the changes of femoral metaphysis trabecular bone after different treatments. In 12 weeks, large areas of defective area still exist in the OVX group, with fewer trabecular bone in the femoral metaphysis. However, a large amount of quantity and maturity trabecular bone were observed filled in the femoral metaphysis from the OVX + HPBC group and the OVX + LPBC group. In comparison, this higher PBC treatment approach can achieve better treatment outcomes, compared with lower PBC treatment at the histological level.

### Immunohistochemical analysis

**Fig. 5:**
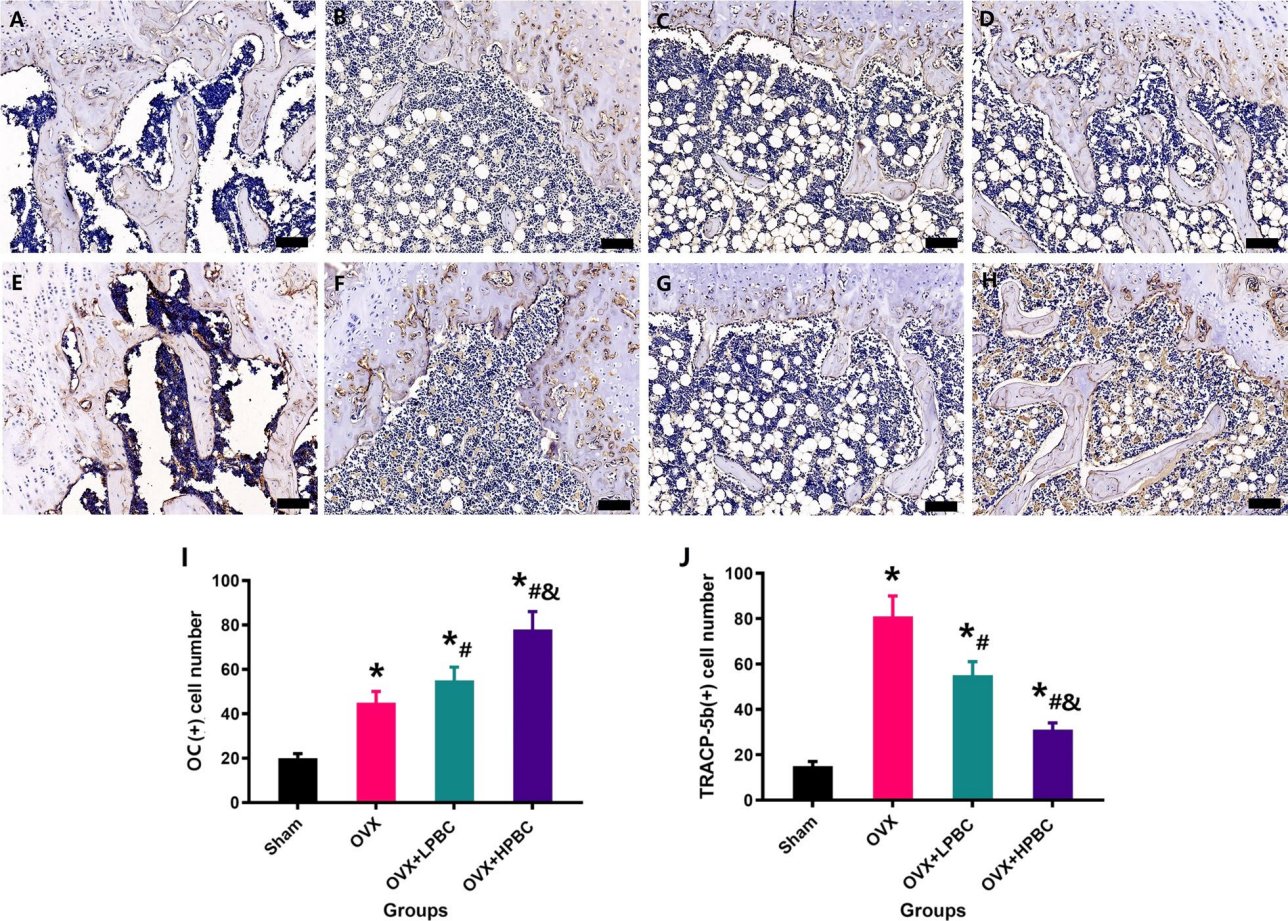
PBC treatment improves the number of osteoblasts and reduces the number of osteoclasts of osteoporosis model rats. After the administration of lower PBC or higher PBC, distal femur was detected by OC staining (**A**–**D**; magnification, × 400) and TRACP-5b staining (**E**–**H**; magnification, × 400). The number of OC-stained osteoblasts was quantified (**I**). The number of TRACP-5b-stained osteoclasts was quantified (**J**). Sham (**A**, **E**), OVX (**B**, **F**), OVX + LPBC (**C**, **G**) and OVX + HPBC (**D**, **H**). n = 5 per group. *Vs. Sham group, P < 0.05, ^#^Vs. OVX, P < 0.05, ^&^Vs. OVX + LPBC group

To determine the underlying mechanisms of PBC’s positive effects, we next performed immunohistochemical analysis of bone tissue. The expression of OC was upregulated in the OVX + LPBC and OVX + HPBC groups. In addition, TRACP-5b staining showed an increase in the number of osteoclasts in OVX rats, which decreased after the administration of PBC; TRACP-5b-positive signals in the OVX + LPBC and OVX + HPBC groups were significantly lower than in the OVX group. Additionally, OC staining and TRACP-5b staining were performed on distal femur, which more comprehensively confirmed that higher PBC treatment can achieve better treatment outcomes (Fig. 5).

### Evaluation of bone metabolism indicators

**Fig. 6:**
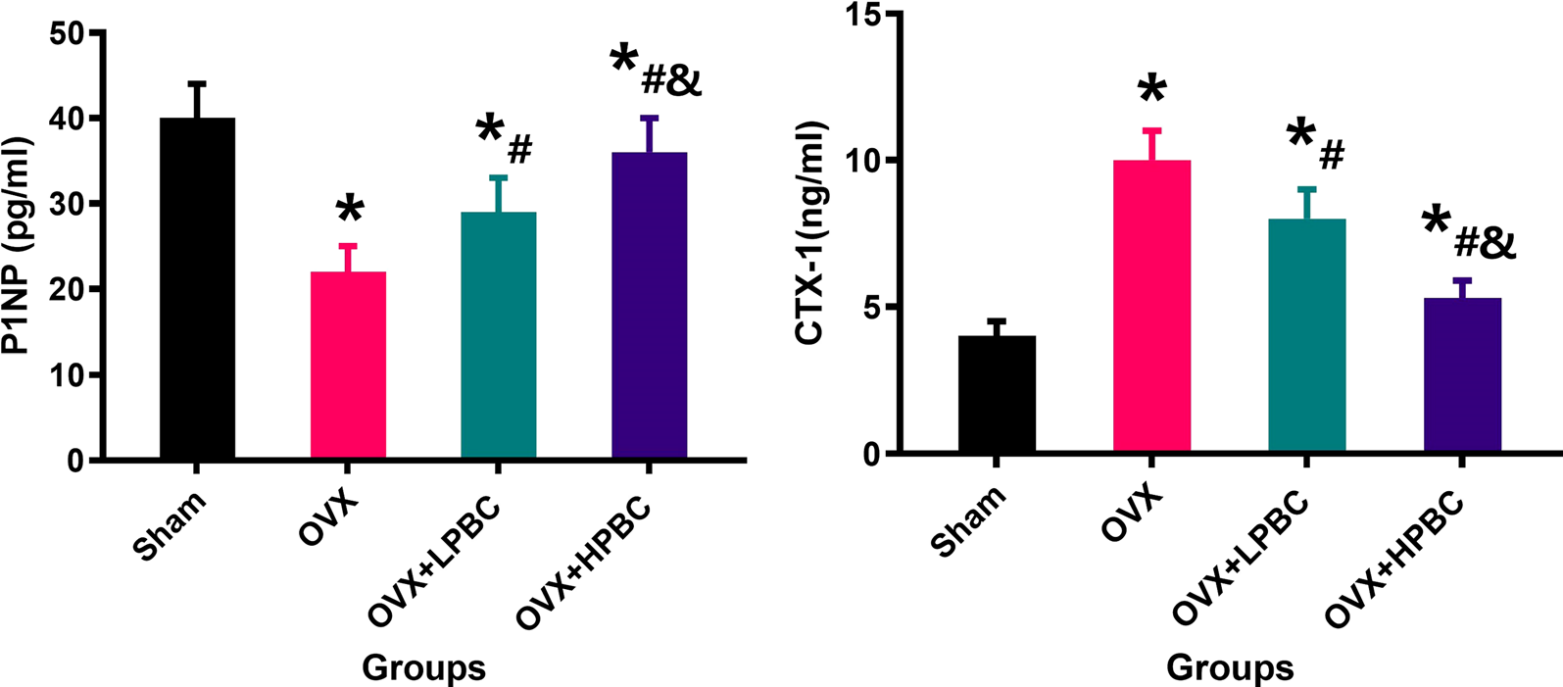
Serum bone metabolism-related indicators P1NP and CTX-1 were determined after the administration of PBC for 12 weeks. n = 5 per group. *Vs. Sham group, P < 0.05, ^#^Vs. OVX, P < 0.05, ^&^Vs. OVX + LPBC group

After treatment with PBC for 12 weeks, serum P1NP and CTX-1 were tested. In the OVX group, the concentration of serum CTX-1 was increased, while P1NP was decreased significantly compared to Sham rats. In both OVX + LPBC group and OVX + HPBC group, these changes were repaired. Bone metabolism testing also showed more obvious changes of P1NP and CTX-1 in the OVX + HPBC rats, compared with lower PBC treatment (Fig. 6).

### The changes of cell viability and function and related genes expression

**Fig. 7:**
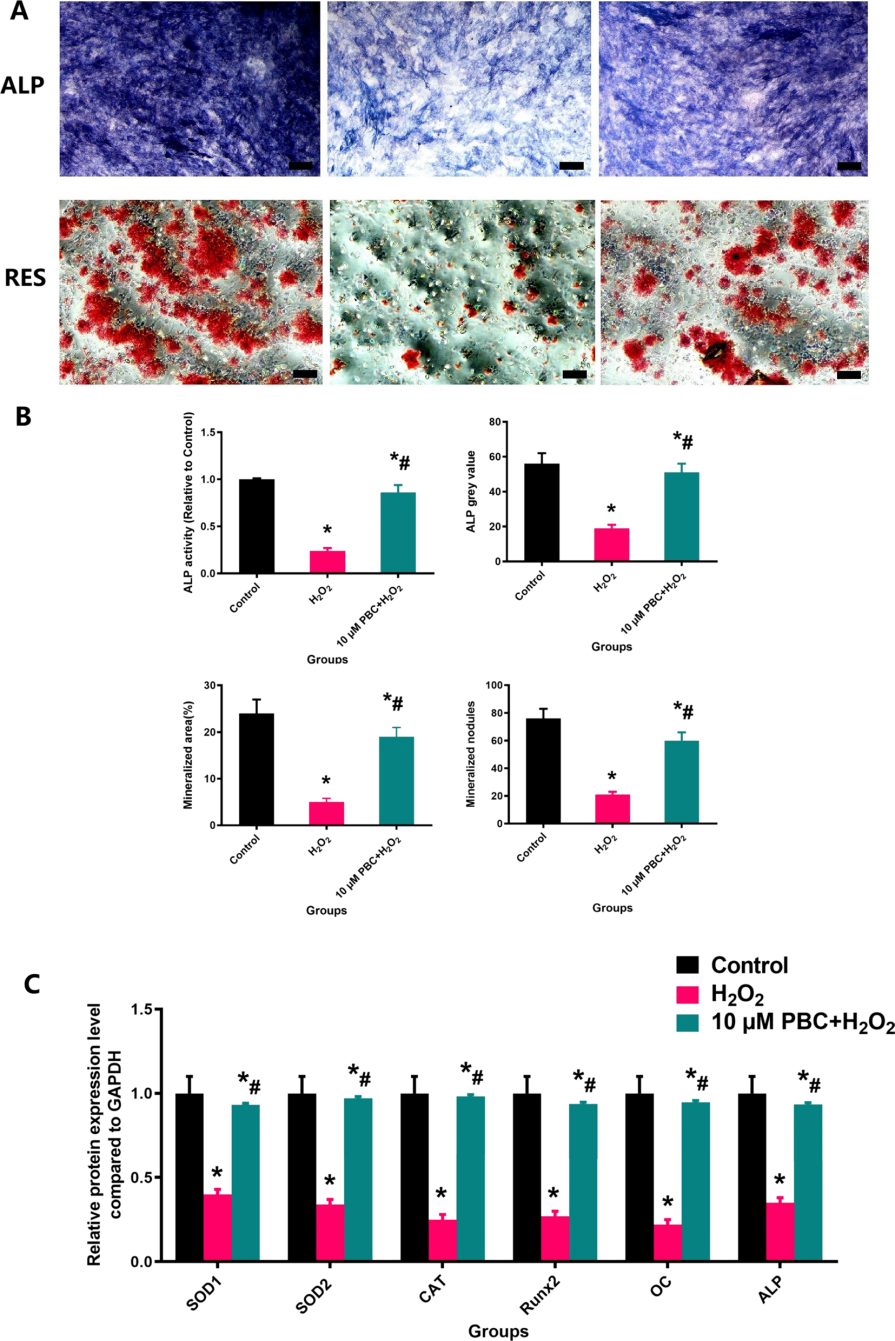
PBC treatment could increase MC3TE-E1 Cells viability, mineralization, ALP expression and related genes expression under H_2_O_2_ intervene. The change of MC3TE-E1 function (**A**), the quantification of mineralized nodules, mineralized area, ALP activity and ALP gray value (**B**) and related genes expression (**C**) after treatment with different intervention options. N = 5 specimens/group. *P < 0.05 vs. Control group, ^#^P < 0.05 vs. H_2_O_2_ group

In order to assess the effect of different intervention options on MC3T3-E1 cells viability and function and related genes expression, this study further conducted Cell Count Kit-8, ALP staining, RES staining and RT-qPCR analysis, as shown in Fig. 7. In the cell experiments, the mineralization and ALP expression of MC3T3-E1 including mineralized nodules (number per well), mineralized area (%), ALP activity and ALP gray value were found to be significantly decreased in the H_2_O_2_ group, while obviously improved in the 10 μm PBC + H_2_O_2_ group. Related genes expressions including SOD1, SOD2, Runx-2, OC, ALP and CAT of the 10 μm PBC + H_2_O_2_ group were significantly lower than that of the H_2_O_2_ group (P < 0.05).

**Fig. 8:**
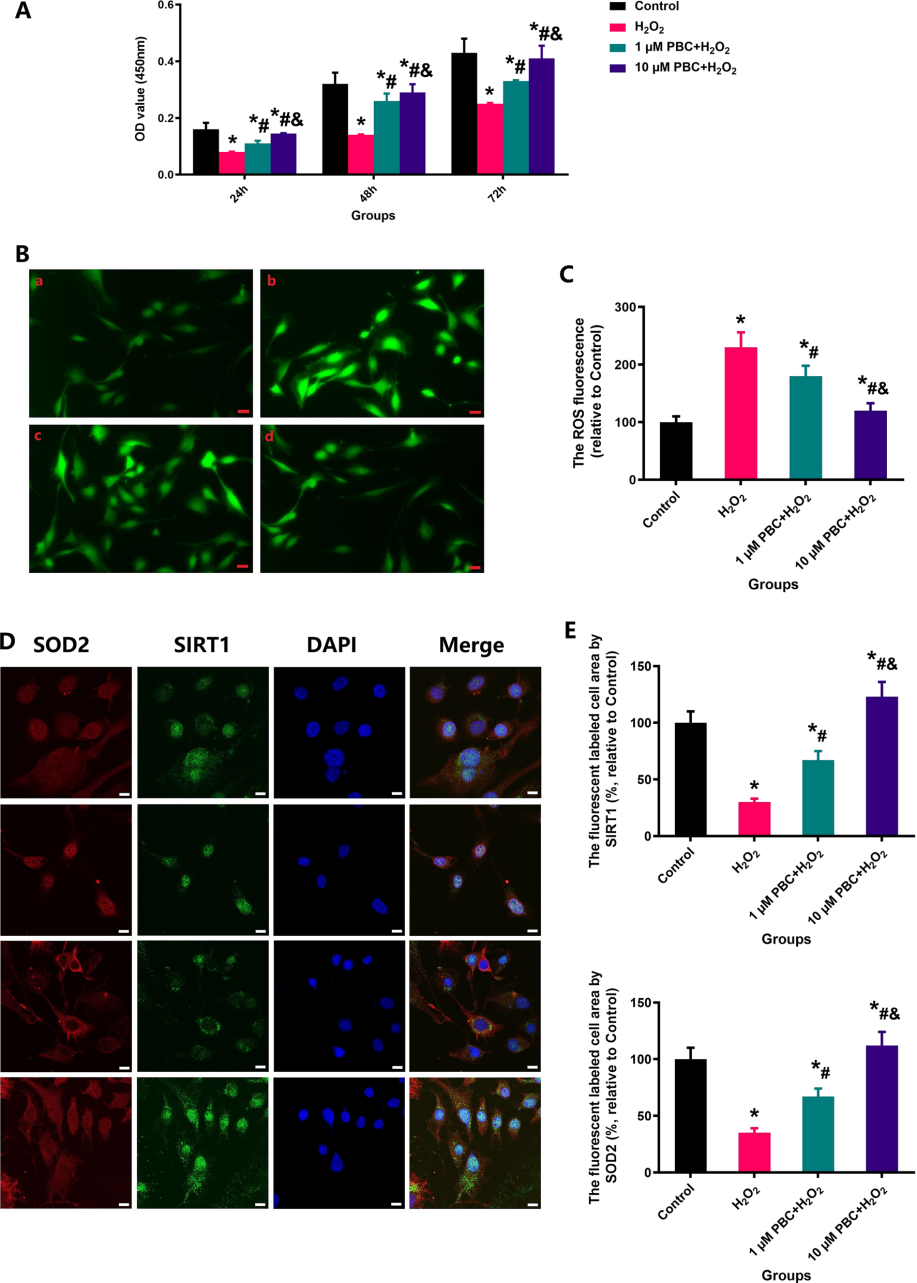
PBC can significantly decrease MC3T3-E1 cellular ROS levels and improve cells viability, the expression of SIRT1 and SOD2 under H_2_O_2_ intervene. Cells viability (**A**), Intracellular ROS level of MC3TE-E1 cells (**B**) and quantitative results (**C**); The changes of SIRT1 and SOD2 protein expression (**D**) and quantitative results (**E**). N = 5 specimens/group. *P < 0.05 vs. Control group, ^#^P < 0.05 vs. H_2_O_2_ group, ^&^P < 0.05 vs. 1 μm PBC + H_2_O_2_ group

Treatment with 10 μm PBC + H_2_O_2_ and 1 μm PBC + H_2_O_2_ could enhance the cell viability at 24 h, 48 h and 72 h (P < 0.05, Fig. 8A). The results of cellular ROS levels are shown in Fig. 8B. ROS levels increased significantly after H_2_O_2_ intervention, while cellular ROS levels significantly decreased after 10 μm PBC + H_2_O_2_ intervention, compared with 1 μm PBC + H_2_O_2_ and H_2_O_2_ groups. The quantitative results are shown in Fig. 8C. ROS was significantly reduced by the intervention of 10 μm PBC + H_2_O_2_ and 1 μm PBC + H_2_O_2_ (P < 0.05). To more intuitively observe the expression of SIRT1 and SOD2, immunofluorescence was performed on the MC3TE-E1 under different intervention actions. The representative immunofluorescence images and quantitative results of SIRT1 and SOD2 expression are shown in Fig. 8D. The expression of SIRT1 and SOD2 decreased significantly after H_2_O_2_ intervention, while the SIRT1 and SOD2 levels significantly increased after 10 μm PBC + H_2_O_2_ intervention, compared with 1 μm PBC + H_2_O_2_ and H_2_O_2_ groups (P < 0.05).

## Discussion

Postmenopausal osteoporosis is a familiar age-linked bone illness and a principal threat to the public health of aged women (Jacobs et al. 2012). Numerous promising pharmacotherapies have been examined as possible treatments for postmenopausal osteoporosis (Feng and McDonald 2011). However, these synthetic drugs are not all patients responding and some even bring severe side effects (Wang et al. 2013). Therefore, it is necessary to develop alternative agents that are safe and effective enough to treat osteoporosis in postmenopausal women. Studies assure that PBC is supposed to protect pulmonary fibrosis via anti-oxidation properties (Zhang et al. 2019). Nevertheless, the mechanism of action of PBC in postmenopausal osteoporosis remains ambiguous. Hence, exploration of the impacts of PBC on OVX rats and MC3T3-E1 cells was conducted, and its mechanism manifested. Here, our experimental results suggested that PBC could enhance osteoblast viability and promote osteoblast differentiation, protect MC3T3-E1 from oxidative stress induced by the exposure to hydrogen peroxide, and significantly protect against OVX-induced osteoporosis by inhibiting oxidative in model rats.

In this study, we first focused on the in vitro effects of PBC on the proliferation and differentiation of osteoblasts, the viability and function of osteoblasts. OC is a strong marker of bone formation and plays a crucial role in osteoblasts differentiation and mineralization at the late stages (Liu et al. 2016). In the early stages of osteogenic differentiation, the expression of RUNX2 is a key factor for regulating osteogenic differentiation and osteoblast activation (Farrokhi et al. 2015; Yu et al. 2018). As an important marker at the early stage of osteogenic differentiation, ALP is an essential enzyme in the construction of bone matrix (Nakamura et al. 2009). Therefore, the expression of ALP mass and OC, and Runx2 gene of osteoblasts were measured by ALP staining and RT-qPCR. In vitro experiments, significant increase in ALP specific activity was observed after 14 days of PBC intervention, as well as highly expressed OC and Runx2. In addition, PBC at 10 µM concentrations protected MC3T3-E1 against hydrogen peroxide-induced reactive oxygen species (ROS) production.

Oxidative stress reflects a state of physiologic imbalance between the production of ROS and the antioxidant network (Chan et al. 2013). When this occurs, oxidation of important macromolecules include DNA, lipids, and proteins (Durgun et al. 2020). Many reports have shown that ROS may be involved in the pathogenesis of bone loss in patients with osteoporosis (Mohamad et al. 2012). Estrogen deficiency and aging have been linked to increased levels of ROS and increase in differentiation of osteoclast precursors, thus resulting in increased bone resorption (Zhan et al. 2019). In addition, overproduction of ROS attenuates osteoblastogenesis and osteocyte lifespan, resulting in limited bone formation. Due to its favorable anti-inflammatory and anti-oxidant properties, probucol could inhibit the initiation and progression of atherosclerosis (Yamashita and Matsuzawa 2009). Thus, we further explored the effects of probucol against oxidative stress induced by hydrogen peroxide in osteoblast and found that probucol significantly promotes the expression of oxidative stress-specific genes, such as SOD1, SOD2 and CTA (Song et al. 2022). Besides, systematic administration of PBC significantly promotes the expression of SIRT1 and SOD2 through immunofluorescence in hydrogen peroxide-treated osteoblasts. These results suggest that probucol plays an important role in osteoblasts viability and differentiation under a rise in ROS levels.

Reductions in circulating estrogen in postmenopausal women can be appropriately mimicked in rodents by bilateral removal of the ovaries (Brommage et al. 2019). Considering more bone volume available for structural and mechanical analysis, distal femurs were selected for easy analysis and testing. Hence, in our animal experiments, we investigated the changes of bone mass and BMD in femoral metaphysis bone after PBC treatment. BMD is used clinically to predict fracture risk and has been used as the gold standard for monitoring changes in bone mass (Farlay et al. 2016). In addition, biochemical markers of bone turnover are classified into two subclasses including bone formation and bone resorption (Diem et al. 2014). Thus, we detected and analyzed the changes in BMD and PINP and CTX in different groups after treatment with probucol. The results in animal experiments are summarized as follows: (i) Notably, the reduced BMD value of OVX rats was reversed in the probucol treatment group; (ii) Further, the levels of CTX were significantly reduced after probucol treatment. The increased PINP levels were observed in the probucol treatment group. Moreover, higher probucol was much more effective in altering these markers than lower probucol; (iii) Probucol treatment clearly preserved trabecular microstructures in vivo. Remarkably, increased OC expression and decreased TRACP-5b expression observed by immunohistochemistry were consistent with altered serum levels of PINP and CTX. In view of the above results, we could conclude that osteoporosis caused by estrogen deficiency can upregulate the level of oxidative stress in osteoblasts, resulting in decreased osteogenic capacity, which is manifested as decreased ALP expression and reduced mineralization function and impaired bone formation capacity, and eventually leads to bone loss in OVX rats. Moreover, the application of probucol can restore the oxidative stress balance of osteoblasts and protect the activity and function of osteoblasts, thereby improving the osteogenic ability and enhancing bone formation in OVX rats.

## Conclusion

In summary, our research results confirm that osteoporosis caused by estrogen deficiency impairs the activity and function of MC3T3-E1 and inhibits bone formation by up-regulating intracellular ROS levels. Probucol can improve the activity and function of osteoblasts and enhance bone formation mediated by reducing intracellular ROS in OVX rat models. However, due to the short experimental time, less experimental animals and lack of positive control group, the shortcomings of this study require further exploration and confirmation.

## Data Availability

All data generated or analyzed during this study are included in this published article.

## Abbreviations

PBC: Probucol
OVX: Ovariectomized
SD: Sprague-Dawley
HE: Hematoxylin and eosin
RT-qPCR: Reverse transcription-quantitative polymerase chain reaction
P1NP: Procollagen type I N-terminal propeptide
CTX: Type I collagen carboxy terminal peptide
VOI: Volume of interest
BMD: Bone mineral density
BV/TV: Bone volume per total volume
Tb.Th: Bone trabecular thickness
Tb.N: Trabecular number
Tb. p: Trabecular spacing
Conn.D: Connective density
TRACP-5b: Tartrate-resistant acid phosphatase type 5 precursor
Runx2: Runt-related transcription factor 2
OC: Osteocalcin
